# Zinc Biofortification through Basal Zinc Supply Reduces Grain Cadmium in Mung Beans: Metal Partitioning and Health Risks Assessment

**DOI:** 10.3390/toxics10110689

**Published:** 2022-11-14

**Authors:** Md Harunur Rashid, Mohammad Mahmudur Rahman, Ravi Naidu

**Affiliations:** 1 Global Centre for Environmental Remediation (GCER), College of Engineering, Science and Environment, The University of Newcastle, Callaghan, NSW 2308, Australia; 2 Cooperative Research Centre for Contamination Assessment and Remediation of the Environment (CRC CARE), ATC Building, The University of Newcastle, Callaghan, NSW 2308, Australia; 3 Bangladesh Agricultural Research Institute (BARI), Gazipur 1701, Bangladesh; 4 Department of General Educational Development, Faculty of Science & Information Technology, Daffodil International University, Dhaka 1207, Bangladesh

**Keywords:** mung beans, zinc, cadmium, partitioning, accumulation, health risk, translocation factor, pore water, correlation

## Abstract

Grain zinc (Zn) biofortification with less cadmium (Cd) accumulation is of paramount importance from human health and environmental point of view. A pot experiment was carried out to determine the influence of Zn and Cd on their accumulations in Mung bean tissues (*Vigna radiata*) in two contrast soil types (Dermosol and Tenosol). The soil types with added Zn and Cd exerted a significant effect on translocation and accumulation of metals in different tissues. The accumulation of Zn and Cd was higher for Tenosol than that for Dermosol. At control, the concentration of Cd followed a pattern, e.g., root > stem > petiole > pod > leaflet > grain for both soils. A basal Zn supply (5 mg kg^−1^) increased the grain Zn concentration to a significant amount (up to 67%). It also reduced Cd accumulation in tissues, including grains (up to 34%). No non-carcinogenic effect was observed for either the children or the adults as the EDI and PTDI values were below the safety limit; however, the ILCR values exceeded the safety limit, indicating the possibility of some carcinogenic effects. Added Zn helped to reduce the carcinogenic and non-carcinogenic health risks on humans.

## 1. Introduction

Cadmium (Cd) is a toxic heavy metal that easily enters the food chain as plants can easily take it up [1,2,3]. Its uptake is varied depending on plant species, cultivars metal concentrations, and types [4,5,6,7,8]. Cd contamination is caused due to both natural and anthropogenic sources, e.g., the industrial activities, mining, the usage of batteries, contaminated waste materials, sludge, and the use of pesticides and phosphate fertilizers etc. [9,10,11]. Studies revealed that it is heavily phytotoxic and inhibits plant growth and caused even death [1,12,13]. On the other hand, zinc (Zn) is an essential nutrient element for both plants and humans. The availability of heavy metals in soils and plants depend on various factors, i.e., soil organic matter, soil pH, cation exchange capacity, etc. [14,15,16,17]. The availability of these metals decreases when the pH of the soil increases [18]. It is evident that soil Zn supply decreased Cd accumulation [19,20,21], which involves soil chemical processes and other factors. A study illustrated the competitive interactions of these metals for their uptake in the existence of a common transport system in plasma membranes [22,23,24,25]. It was reported that soil Zn supply reduced the accumulation of Cd in crops [26,27]. On the contrary, another study demonstrated that Cd concentration increased with Zn concentration in wheat or vice versa [4,5,28,29]. An inconsistent relationship between Zn and Cd on the uptake in roots and leaves of lettuce and spinach was also reported [30]. Wu and Zhang [31] stated that the Zn supply alleviated the effect of Cd stress in barley through enhanced growth and reduction of membrane damage. Research showed that the translocation of Cd and Zn into mustard and to seeds as a role of exogenous Cd and the stage of the life cycle (vegetative growth to seed set) determined the critical developmental windows where the translocation from the roots to seeds was the greatest [32]. Plants avert Cd toxicity and accumulation through various detoxification mechanisms. One of the processes is sequestration in root vacuoles, chelation, the induction of antioxidant machinery, and hormonal regulation which limits its translocation to other parts, especially edible ones [33,34,35,36,37]. In mature plant cells, these vacuoles are the largest organelle that acts as a reservoir of ions and metabolites that are crucial to detoxifying Cd and for normal cell development [38,39]. Studies with Zn-Cd interactions were performed on mostly cereals and a few on vegetables where only selected tissues were considered and analyzed. Being an important pulse crop, the mung bean was not examined for Zn-Cd interactions. Moreover, analyzing only the selective tissue did not demonstrate the total scenario for the metal translocation patterns to the edible portion of crops. It is important to determine how metals move upward in plants grown in contrasting soil conditions to facilitate the efficient agricultural and environmental management of mung beans with low Cd and high Zn contents in grains. Accordingly, we hypothesized that differences in the adsorption and allocation to plant parts are related to differences in the tissue accumulation of metals. We investigated this hypothesis by conducting pot experiments with two contrast soils (Dermosol and Tenosol); to be specific, we compared Zn and Cd concentrations, their translocations, and partitioning in various plant segments along with their correlations within tissues.

## 2. Materials and Methods

### 2.1. Soil Collection, Processing, Analysis, and Experimental Design

Two types of soils (Dermosol and Tenosol) were used for the experiment, one was silty loam, collected from Taree (32°87′58.3” S, 151°64′00.6” E), and the other was Sandy loam, collected from Newcastle (32°52′34.7” S, 151°38′15.8” E) located in NSW, Australia. The surface soil was air-dried, crushed, cleaned by removing plant debris, pebbles, weeds, stones, etc., and sieved through a 4 mm mesh. A portion of the processed soils was further sieved with 2 mm of mesh for chemical analysis. The physico-chemical properties of the experimental soils are given in Table 1. The texture of the soils was determined according to the hydrometer method [40]. Soil pH was determined with a pH meter (Laqua, Horiba Scientific, Piscataway, NJ, USA), and CEC was measured using the BaCl_2_ compulsive exchange method [41]. To measure other metals/metalloids (Cu, Mn, Zn, Mo, Pb Cd, Cr, Ni, Se, and As), a portion of the soil samples (0.5 g) were digested in a microwave digestion system (MARS 6 240/50, USA), and elements were extracted using 5 mL aqua regia by inductively coupled plasma optical emission spectrometry (ICP-OES, Model: Avio 200, PerkinElmer Pvt. Ltd., Singapore). DTPA-Zn (Diethyltriamine Pentaacetic Acid Extractable Zn) was measured by extracting the soil samples with 0.0005 mol L^−1^ DTPA in 0.01 mol L^−1^ CaCl_2_ buffered at pH 7.3 with 0.1 mol L^−1^ triethanolamine at a soil/solution ratio of 1:2 [42]. Each of the soil samples was measured in triplicate.

The soils were spiked with Cd as cadmium nitrate: the control that contained no Cd and spiked that consisted of 2 and 4 mg Cd kg^−1^ soil. Soils were incubated for three months. Based on the concentration of Cd in the agricultural soils [43,44,45], the levels of Cd in this study were chosen. After acclimatization with Cd, the soils were amended with four levels of Zn as a zinc sulfate heptahydrate: the control (no Zn), 5, 10, and 20 mg Zn kg^−1^ of soil. The soils in each pot were fertilized with 30 mg N kg^−1^ of soil as urea, 50 mg P kg^−1^ of soil as a single super phosphate, and 30 mg K kg^−1^ of soil as potassium chloride. The full amount of P and K and half of N were applied to the soil and mixed before sowing, and the other half of N was applied in two equal splits at 30 and 45 days after sowing. The treated soils were mixed thoroughly, put into plastic pots (4.0 kg pot^−1^), and saturated with deionized water (18.2 MΏ cm). Each treatment (number of soils: 2; the number of Zn levels: 4 and number of Cd levels: 3; total treatments: 24) was replicated three times.

### 2.2. Plant Growth

The plastic pots were randomly placed in the greenhouse, which was further rearranged after every 3 days during the growth period for an even distribution of sunlight. Seedlings were thinned to two per pot when they were about 4 cm in height. The seedlings were irrigated with deionized water (18.2 MΏ cm) to maintain 70% of the field capacity of the soil. Plants were cultivated at 25/20^0^C day/night temperature, 75% relative humidity, 16/8 h photoperiod. Seeds of mung beans of the variety Jade-AU were obtained from the Australian Mung bean Association (AMA) and used as experimental material. The variety was moderately weather resistant, lodging resistant, fairly susceptible to powdery mildew, tan spot, and halo blight, tall and with a 100-grain weight of 6–7.3 g [46]. Plastic pots with a 24 cm internal diameter and 30 cm height were filled with 4 kg of soil.

3% hydrogen peroxide (*v*/*v*) was used to sterilize the seeds followed by a rinse with deionized water. After soaking in deionized water for about six hours, ten seeds were placed per pot and covered with soft disposable tissue paper. Until germination, the tissue paper was kept moist with water spray to protect it from heat and retain moisture. After germination, the seeds were covered with a thin layer of soil.

### 2.3. Plant Harvest and Analysis

Plants were harvested at maturity. Roots and shoots were separated immediately, washed with deionized water, and dried in an oven (65 °C) until they attained a constant weight. Dry biomass was taken. The shoots were further divided into five parts (stem, petiole, leaflet, pod, and grain) to determine the accumulation of the metals in different plant parts. A stainless-steel grinder was used to grind the samples. To digest all plant samples, a microwave digester (CEM, MARS 6) along with 40 digester vessels was used. About 0.25 g of finely ground samples were taken into a Teflon vessel where 3 mL of concentrated HNO_3_ and 2 mL of H_2_O_2_ were added. Prior to digestion, the samples were kept overnight (16 h) in a fume hood. Each vessel was sealed and placed into the rotor. Each digestion solution was transferred to a plastic tube and diluted to 10 mL using ultrapure water (ELGA PureLab, USA). Samples were filtered through syringe filters (MCE 0.45 μm) and analyzed on the same day of preparation, otherwise, they were stored in a fridge at 4 °C. The metal concentrations (Zn and Cd) in the digested samples were measured by inductively coupled plasma mass spectrometry (ICP-MS, Agilent 7900, Japan). Reagent blank, duplicates, continuing calibration verification (CCV), and spinach leaves as a standard reference material (SRM 1573a) collected from the National Institute of Standards and Technology (NIST) were used (*n* = 60) to verify the results. Spinach leaves were digested using the same procedure. The recovery percentage of the SRM was about 91% and 92% for Zn and Cd, respectively.

### 2.4. Soil Pore Water Analysis

Porewater from the soils of each pot was collected at four times e.g., 15, 30, 45, and 60 days after sowing (DAS). Rhizon soil pore water sampler (Rhizosphere Research Products, The Netherlands) was used for the collection. The Rhizon samplers were vertically inserted into the puddled soil up to a depth of 10 cm. A syringe (10 mL) was used to collect the pore water. After collection it was then filtered (0.45 µm), and acidified with high-purity nitric acid (7 M) [47] for analysis with ICP-MS.

### 2.5. Human Health Risks Assessment

The potential human health risks were assessed from Cd exposure via the consumption of pulses. The estimated daily intake (EDI) of Cd, target hazard quotient (THQ), and incremental lifetime cancer risk (ILCR) were determined.

#### 2.5.1. Measurement of Daily Intake of Heavy Metals

Estimation of human health risks was accomplished using the estimated daily intake (EDI) values followed by a comparison with the maximum tolerable daily intake (MTDI) set by regulatory bodies. The following equation was used for the determination [48,49,50], based on the US EPA:

(1) $$\mathrm{EDI}=\frac{IngR\times C}{BW}$$

where EDI = estimated daily intake of Cd from pulses consumed (mg d^−1^) per BW (kg), *IngR* = ingestion rate (kg) of pulses in Bangladesh, *C* = concentration of Cd in the sample (mg kg^−1^), and *BW* = body weight (kg). The average pulses consumption rate worldwide (0.021 kg person^−1^ day^−1^) [51] and BW values for adults (71.8 kg) and children (32 kg), respectively [52,53].

#### 2.5.2. Non-Carcinogenic Health Risk

Target hazard quotient (THQ) was estimated to ascertain the non-carcinogenic risks of Cd in the sample [49,54]:

(2) $$\mathrm{THQ}=\frac{\mathrm{EDI}}{RfD}$$

where *R_f_D* = oral reference dose (1 × 10^−3^ mg kg^−1^ daily for Cd) [55].

#### 2.5.3. Carcinogenic Risk

Incremental lifetime cancer risk (ILCR) was calculated to ascertain the possibility of cancer risk for the intake of Cd via mung beans [56]:

ILCR = EDI × CSF(3)

where CSF = cancer slope factor (6.3 mg kg^−1^ per day) [57,58].

### 2.6. Statistical Analysis

JMP Pro 14.2.0 software was used for statistical analysis. Mean comparison was done by two-way ANOVA using Student’s *t*-test at *p* < 0.01 or *p* < 0.05. Figures were prepared by GraphPad Prism Software (version 9.0.0, GraphPad Software, Inc. San Diego, CA, USA). Standard errors (SE) were calculated (*n* = 4) using Microsoft Excel.

## 3. Results

### 3.1. Partitioning of Cadmium and Zinc in Mung Beans

**Figure 1:**
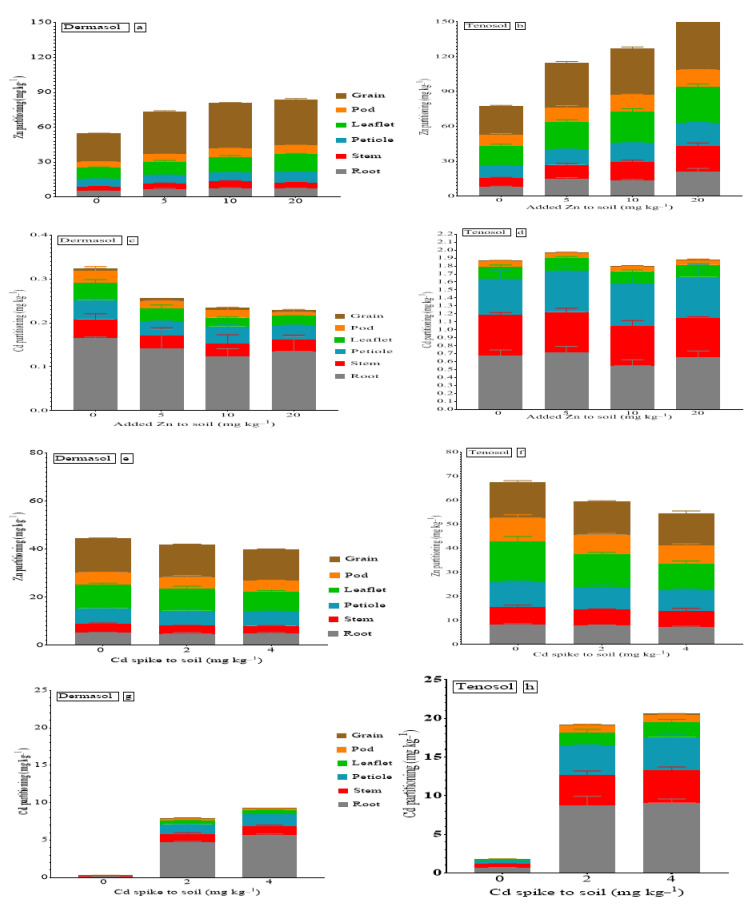
(**a**–**h**) Partitioning of Zn and Cd in mung beans as affected by Zn supply, Cd, and soil types.

**Figure 2:**
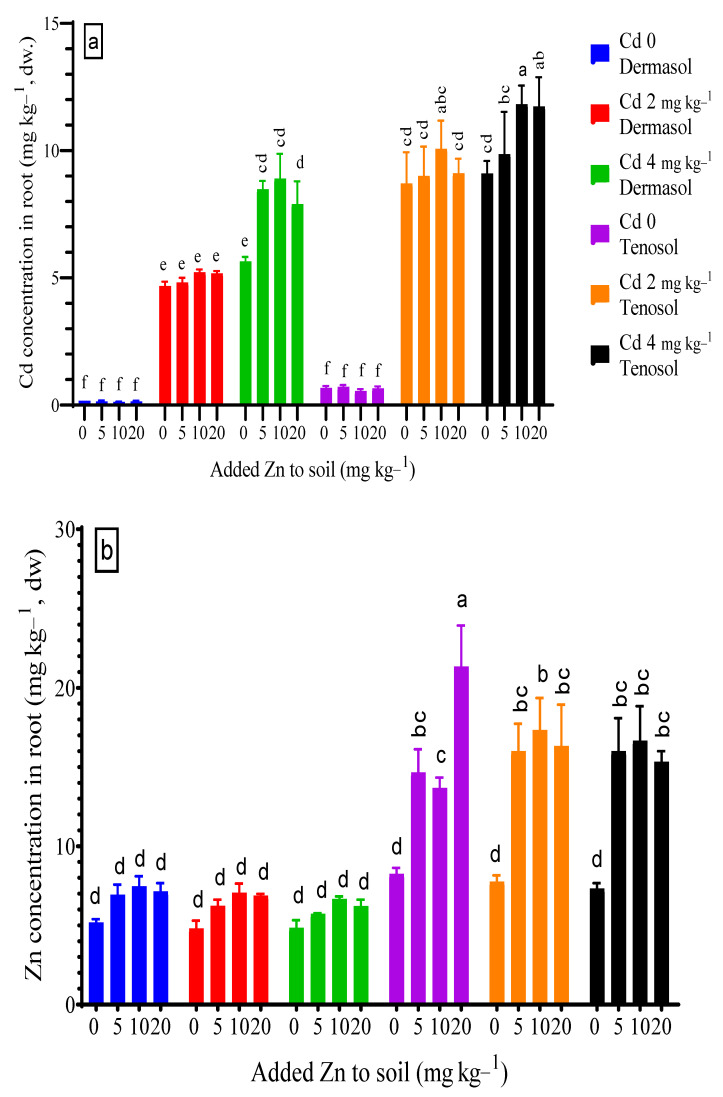
(**a**,**b**) Concentrations of Cd and Zn in roots as affected by added Zn and Cd contamination in soils. Values with a common letter do not differ significantly (*p* < 0.05).

**Figure 3:**
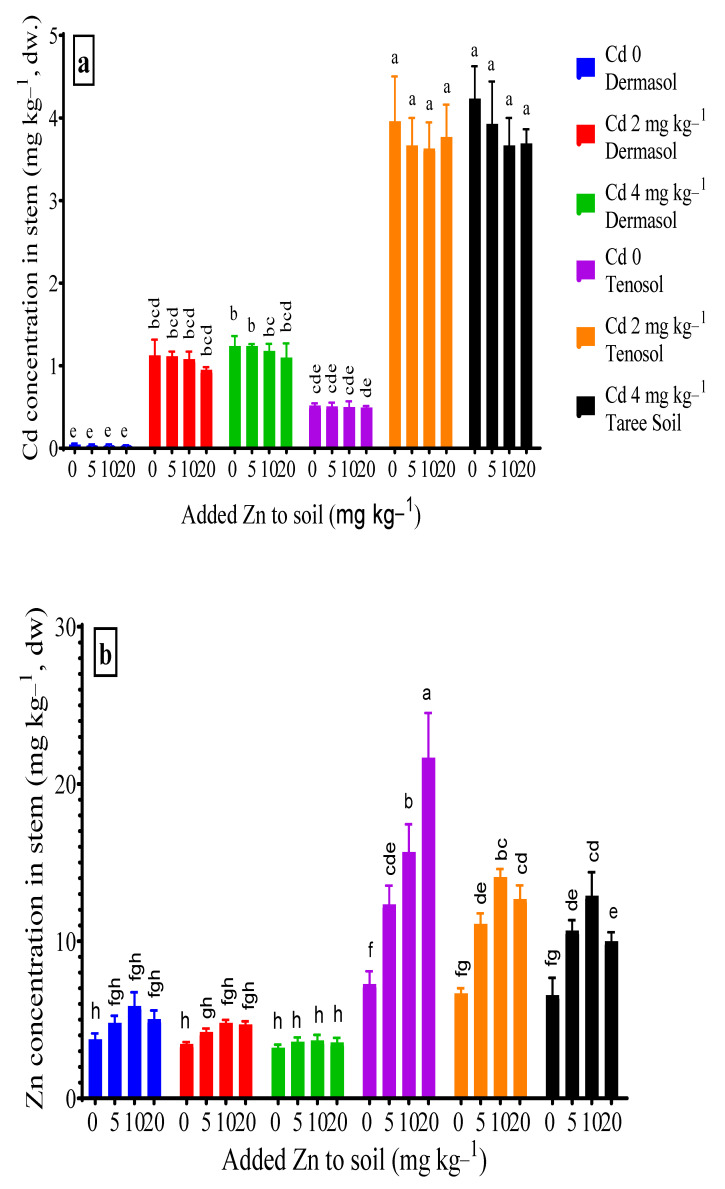
(**a**,**b**) Concentrations of Cd and Zn in stem as affected by added Zn and Cd contamination in soils. Values with a common letter do not differ significantly (*p* < 0.05).

**Figure 4:**
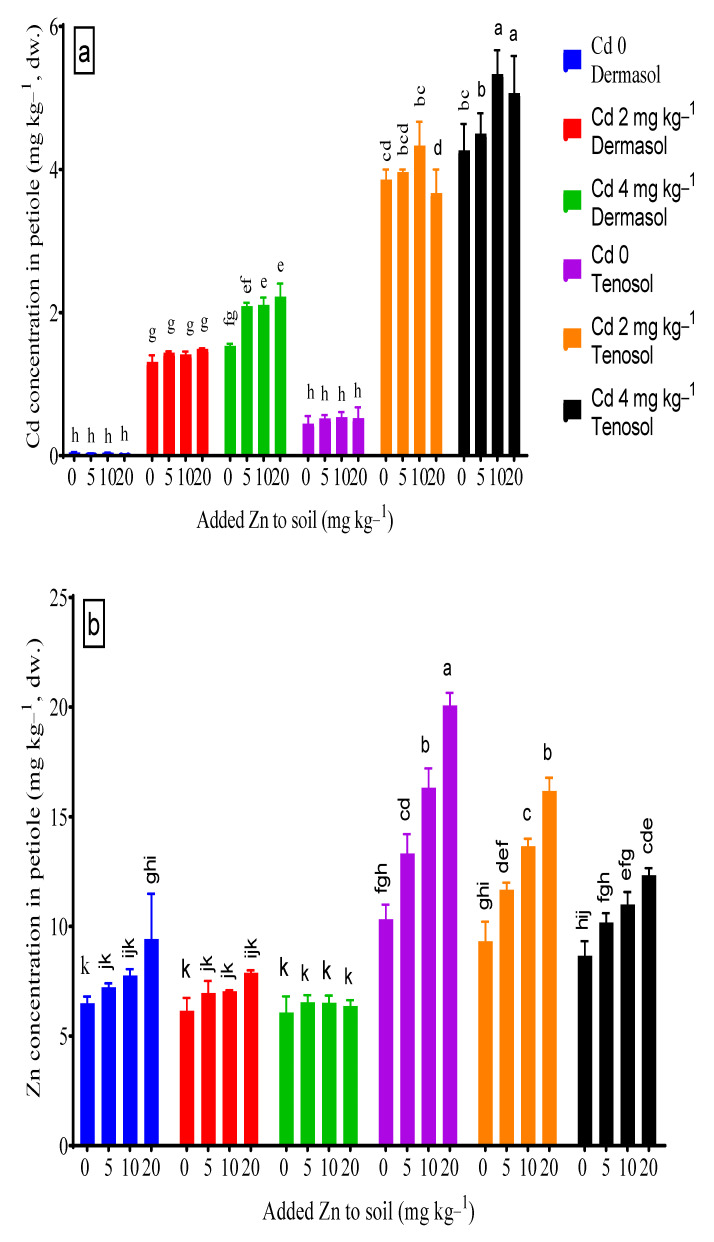
(**a**,**b**) Concentrations of Cd and Zn in petiole as affected by added Zn and Cd contamination in soils. Values with a common letter do not differ significantly (*p* < 0.05).

**Figure 5:**
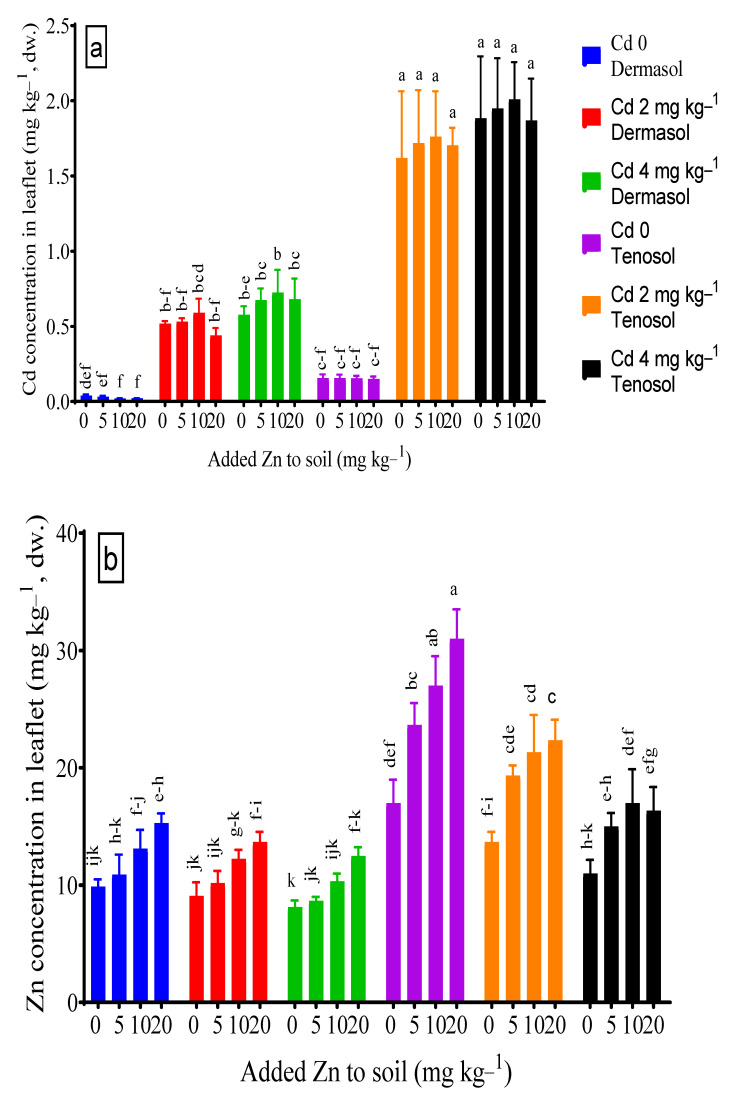
(**a**,**b**) Concentrations of Cd and Zn in leaflet as affected by added Zn and Cd contamination in soils. Values with a common letter do not differ significantly (*p* < 0.05).

**Figure 6:**
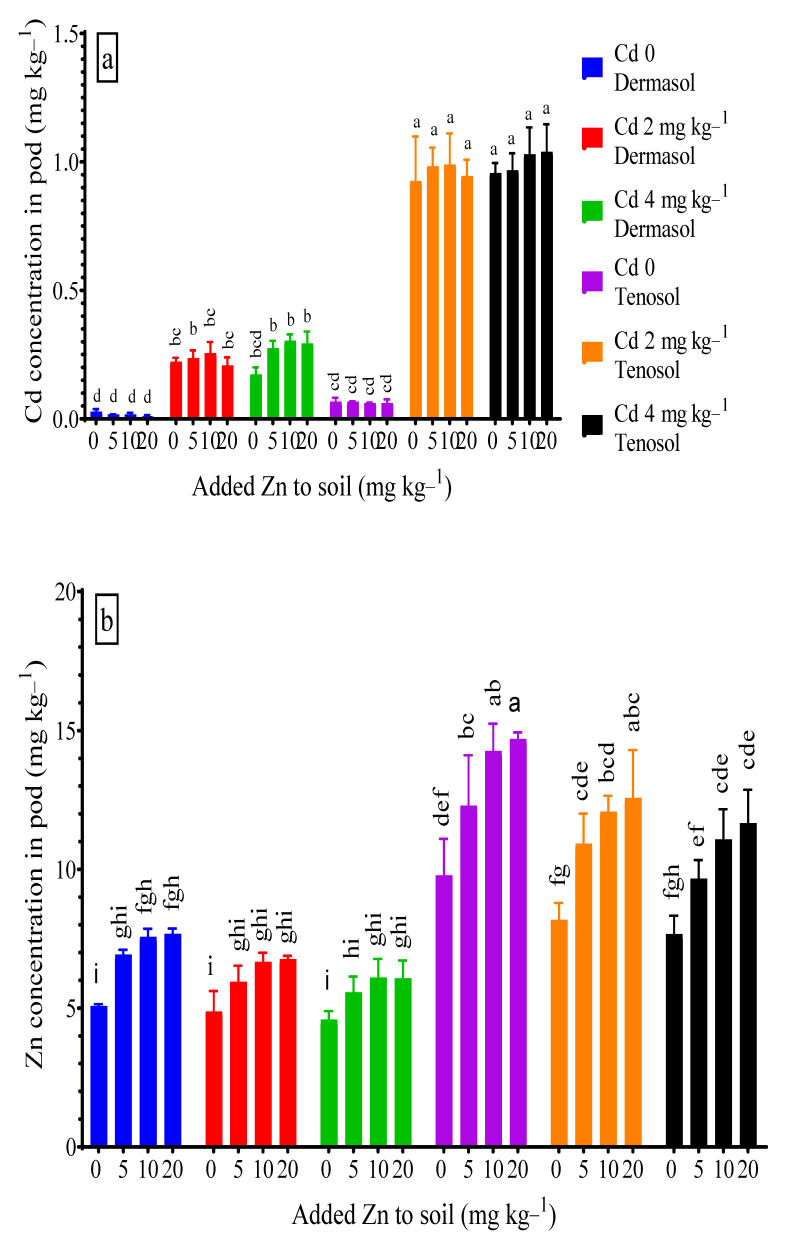
(**a**,**b**) Concentrations of Cd and Zn in pod as affected by added Zn and Cd contamination in soils. Values with a common letter do not differ significantly (*p* < 0.05).

**Figure 7:**
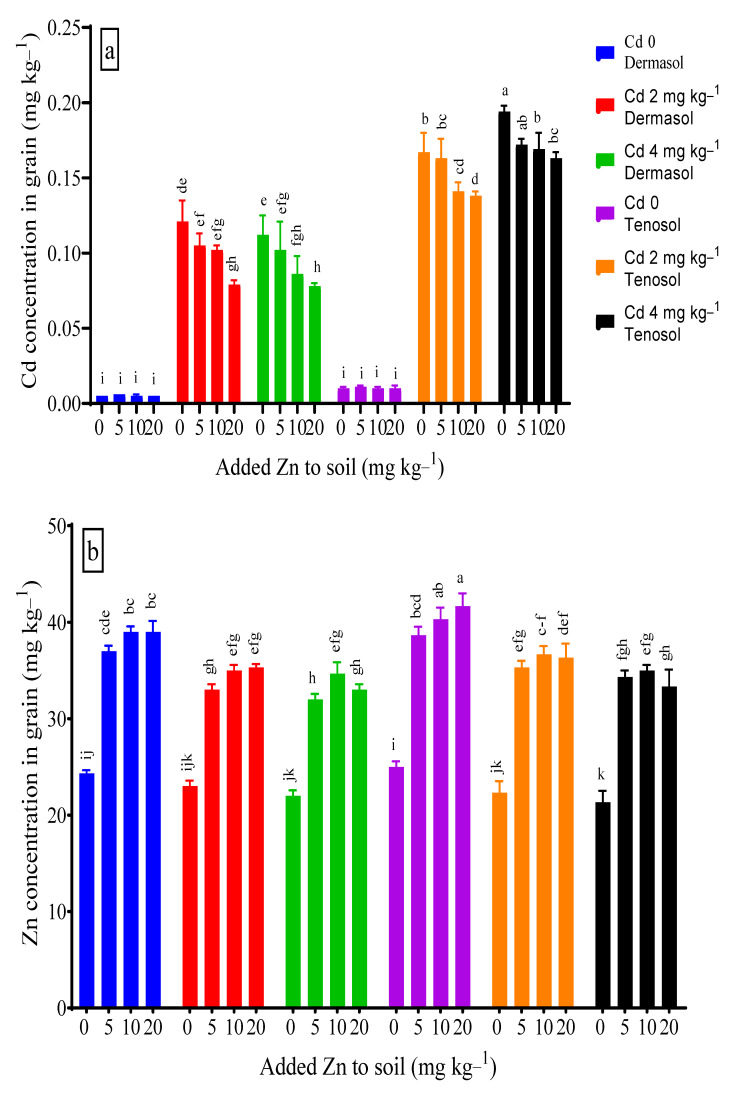
(**a**,**b**) Concentrations of Cd and Zn in grains affected by added Zn and Cd contamination in soils. Values with a common letter do not differ significantly (*p* < 0.05).

All the measured parameters differed based on Cd contamination, Zn supply, and soil types. The concentrations of six parts (root, stem, petiole, leaflet, pod, and grain) derived from mung bean plants were analyzed for Cd and Zn concentration, partitioned, and are presented in Figure 1. The concentration of Zn followed the order gain > leaflet > petiole > pod > root > stem (Figure 1a) and grain > leaflet > petiole > stem > root > pod (Figure 1b) for Dermosol and Tenosol, respectively, because of the added Zn. On the other hand, Cd contamination altered the order of the Zn concentration in the tissues for Tenosol (leaflet > grain > petiole > pod > root > stem) (Figure 1f), while Dermosol demonstrated the same partitioning (Figure 1e). Cd partitioning due to the added Zn distinctly differed for both soils (Figure 1c,d). The difference in the partitioning of Cd was also prominent for the soils as far as Cd contamination was concerned (Figure 1g,h). Each partitioning of the mung beans is separately described in Figure 2, Figure 3, Figure 4, Figure 5, Figure 6 and Figure 7 as influenced by the basal Zn supply, Cd contamination, and soil types.

### 3.2. Concentration of Cadmium and Zinc in Roots

The Cd concentration in the roots was significantly affected by the soil (*p* < 0.01), basal Zn supply (*p* < 0.05), and Cd contamination (*p* < 0.01). Irrespective of the treatments employed, Tenosol demonstrated a significantly higher concentration of Cd in the root than Dermosol. The contamination of Cd at 2 and 4 mg kg^−1^ to the Dermosol skyrocketed Cd concentration in the roots, such as 27-folds (4.68 mg kg^−1^) and 33-folds (5.65 mg kg^−1^, respectively), which were about 12-folds for Tenosol. At the Cd control, root Cd concentration decreased because of the Zn supply, but this decline was not significant (Figure 2a). Zn concentration in the roots varied significantly (*p* < 0.01) by the soils, added Zn, and Cd contamination. The concentration of Zn rose to as high as 7.5 mg kg^−1^ (44.1% higher) for the added Zn from 5.2 mg kg^−1^ (control) for Dermosol, although the increment was not significant (Figure 2b). On the other hand, a sharp and significant increase (up to 158% higher) in root Zn concentration was demonstrated for Dermosol. The spike of Cd at 2 and 4 mg kg^−1^ to Dermosol caused a decline in the root Zn concentration by about 7.3% and 6.2%, respectively, and about 5.9% and 5.8% for the Tenosol.

### 3.3. Concentrations of Cadmium and Zinc in Stem

Soil types, Cd contamination individually, and their interactions were the significant factors (*p* < 0.01) for stem Cd concentration. For the controls, as well as all the treatments, stem Cd concentrations for the Tenosol were significantly higher (about 12 X). In Dermosol, about 26 X (1.13 mg kg^−1^) and 28 X (1.24 mg kg^−1^) higher Cd accumulated in the stem as compared to control (0.042 mg kg^−1^) because of the added of 2 and 4 mg Cd kg^−1^ to the soils, respectively. By contrast, the increments were about 13 X (7.29 mg kg^−1^) and 16 X (8.84 mg kg^−1^) in Tenosol (Figure 3a). The mean effect of Zn reduced the stem Cd concentration, and no significant difference among the Zn levels was evident. Interactions between the Zn and Cd levels reduced the stem Cd concentration, which was more prominent for Dermosol than Tenosol. The maximum reduction of the stem Cd concentration was observed more in the Dermosol (30%) than the Tenosol (13%) at the interaction between 10 mg Zn kg^−1^ and 2 mg Cd kg^−1^. However, the interaction between Zn and the higher and lower Cd levels was significantly different for the Tenosol. Soil types, added Zn and Cd contamination were the significant factors (*p* < 0.01) that shaped the accumulation of Zn in the stem. At the controls, as well as in all the treatments applied, Tenosol was favored significantly higher for the Zn accumulation in the stem. Added Zn increased the stem Zn concentration up to 56.7% (5.9 mg kg^−1^) for Dermosol, which was virtually tripled (21.7 mg kg^−1^) and for Tenosol compared to the respective control. For Dermosol, the stem Zn concentration fell to 3.2 mg kg^−1^ (14.4%) from 3.8 mg kg^−1^ due to the presence of Cd, whereas the reduction reached as much as 9.6% (6.6%) for the Tenosol (Figure 3b).

### 3.4. Concentrations of Cadmium and Zinc in Petiole

Concentration of Cd in petiole was greatly influenced by the soil types (*p* < 0.01), Zn supply (*p* < 0.05), Cd toxicity (*p* < 0.01), and the interaction between the soil and Cd (*p* < 0.01). At the control, Tenosol accumulated about 10 X higher Cd (0.447 mg kg^−1^) in the petiole than that of Dermosol (0.044 mg kg^−1^). The contamination of Cd at 2 and 4 mg kg^−1^ to Dermosol increased the petiole Cd concentration by about 29 X (1.31 mg kg^−1^) and 34 X (1.53 mg kg^−1^), respectively, in comparison to the control (0.044 mg kg^−1^), whereas the same Cd levels increased about 7.6 X (3.86 mg kg^−1^) and 8.6 X (4.27 mg kg^−1^) for the Tenosol. However, the two Cd toxicity levels revealed no significant differences for Dermosol, but there was a significant difference for Tenosol. The increment of Zn supply to the soils gradually decreased the petiole Cd concentration by up to 30.3% for Dermosol but rose to about 19.7% for Tenosol. There was no significant difference in the petiole Cd concentration among the Zn levels. The Zn supply at 20 mg kg^−1^ to Dermosol spiked with 2 mg Cd kg^−1^ and 4 mg Cd kg^−1^ and was enhanced by about 14% and 40% higher petiole Cd; these amounts were about 12.3% and 18.7% higher for Tenosol, respectively (Figure 4a). Zn concentration in the petiole was significantly (*p* < 0.01) affected by the soil, added Zn, spike with Cd, and their interactions. At the control, Tenosol accumulated almost double the amount of Zn (10.33 mg kg^−1^) than the Dermosol (6.49 mg kg^−1^). The Zn supply to the soils gradually increased the petiole Zn concentration up to 45.2% and 94.3% for Dermosol and Tenosol, respectively. The presence of Cd at 2 and 4 mg kg^−1^ to Dermosol decreased the petiole Zn concentration by about 5.1% (6.16 mg kg^−1^) and 6.5% (6.1 mg kg^−1^), respectively as compared to the control (6.49 mg kg^−1^). Conversely, the same Cd levels diminished by about 9.7% (9.33 mg kg^−1^) and 16.1% (8.7 mg kg^−1^) for the Tenosol (Figure 4b).

### 3.5. Concentrations of Cadmium and Zinc in Leaflets

The soil types and Cd both solely and in combination influenced Cd accumulation in the leaflet significantly (*p* < 0.01). There was no significant effect of the Zn application on the Cd concentration in the leaflet, although a slight decline was observed for both soils. At the control, leaflet Cd accumulation was about 4 X higher in Tenosol (0.155 mg kg^−1^) compared to Dermosol (0.039 mg kg^−1^). Irrespective of Zn and Cd addition to the soil, the concentration of Cd in the leaflet was demonstrably much higher in the Tenosol than in the Dermosol. An amount of 2 and 4 mg Cd kg^−1^ in the soils did not show any significant difference for leaflet Cd in the soils. The application of sole Zn slightly reduced the leaf Cd concentration, although the reduction was more prominent in Dermosol than Tenosol. The added Zn in the soils contaminated with Cd did not restrict Cd accumulation in the leaflet (Figure 5a). Zn concentration in the leaflet varied depending on the soil and added Zn and Cd contamination significantly (*p* < 0.01). At the control, the concentration of Zn in the leaflet basically doubled (17.0 mg kg^−1^) for the Tenosol unlike the Dermosol (9.9 mg kg^−1^). The accumulation of Zn in the leaflet decreased due to the presence of Cd for both the soils, wherein the Tenosol highlighted a larger decline (up to 35.3%) than that of the Dermosol (up to 17.6%) (Figure 5b).

### 3.6. Concentration of Cadmium and Zinc in Pods

The Cd concentration in the pod significantly (*p* < 0.01) varied with the soil types, Cd, and with their interaction. At the control, the Tenosol favored the double accumulation of Cd in the pods. For Dermosol, while it was spiked with 2 mg Cd kg^−1^, the pod Cd soared up to 0.223 mg kg^−1^, which was about 7 X more than control (0.028 mg kg^−1^). The pod Cd concentration slightly declined to 0.173 mg kg^−1^ with a higher Cd level (4 mg kg^−1^). On the other hand, about 13 X higher pod Cd concentration was observed for the Tenosol when it was spiked with Cd; there was no significant difference in the pod Cd when compared with the two Cd treatments. Sole Zn application reduced the pod Cd concentration for both soils but when compared to the respective controls, the reduction due to raised Zn levels was insignificant. Interactions between Zn and Cd in the soils enhanced the pod Cd concentration compared to a sole Cd application (Figure 6a). Zn accumulation in the pods profoundly (*p* < 0.01) varied with the soil types, added Zn, and Cd contamination. Pod Zn concentration doubled for the Tenosol (9.78 mg kg^−1^) as compared to the Dermosol (5.07 mg kg^−1^) at the control. Zn application increased the pod Zn concentration for both soils, which was more than 50% higher for the Tenosol and Dermosol compared to their respective controls. The pod Zn concentration declined when the soils were spiked with Cd, which was very low (up to 9.5%) for Dermosol and higher (up to 21.6%) for Tenosol (Figure 6b).

### 3.7. Concentrations of Cadmium and Zinc in Grains

The grain Cd concentration was significantly (*p* < 0.01) influenced by the soil types, added Zn, and Cd contamination. At the control, grain Cd concentrations (mg kg^−1^) were 0.005 and 0.01 for Dermosol and Tenosol, respectively. As predicted, Cd treatments to the soils at 2 and 4 mg kg^−1^ soared the Cd concentration, with values reaching about 23 X (0.121 mg kg^−1^) and 22 X (0.112 mg kg^−1^) higher for Dermosol and 16 X (0.167 mg kg^−1^) and 19 X (0.194 mg kg^−1^) higher for Tenosol, respectively, in contrast to the respective controls. In Cd-treated soils, Zn showed its antagonistic effect on grain Cd. For Dermosol, about 34% and 31% reductions in the grain Cd were observed for the lower (Cd 2 mg kg^−1^) and higher (Cd 4 mg kg^−1^) Cd spiked soils, respectively, with 20 mg Zn kg^−1^. Conversely, for the Tenosol, the reductions in the grain Cd were less prominent than the Dermosol, these amounts being 17% and 16% due to the interactions with a lower and higher Cd with Zn 20 mg kg^−1^. The added Zn at 5 and 10 mg kg^−1^ reduced the grain Cd concentration, but the effects were statistically identical for both soil types (Figure 7a).

Zn accumulation to the grains was significantly (*p* < 0.01) enhanced by the soil types, added Zn, and Cd contamination. Similar to other tissues, the grain Zn concentration was slightly higher (24.95 mg kg^−1^) for the Tenosol than the Dermosol (24.33 mg kg^−1^) in the control, which were statistically similar. The presence of Cd at 2 and 4 mg kg^−1^ in the soils diminished the grain Zn accumulation by 5.5% and 9.6% for the Dermosol and 11.1% and 14.7% for the Tenosol, respectively. Compared to the controls, adding 5 mg Zn kg^−1^ to Dermosol and Tenosol enhanced the grain Zn accumulation by about 52.1% (37.04 mg kg^−1^) and 54.7% (38.70 mg kg^−1^), respectively. The further addition of Zn increased the grain Zn accumulation with maximum values of 39.03 mg kg^−1^ and 41.7 mg kg^−1^ for Dermosol and Tenosol, respectively. However, there was no significant difference among the added Zn levels. Although a slight decrease in grain Zn was observed for both soils, the added Zn ensured an upward Zn accumulation in the grains (Figure 7b).

### 3.8. Correlation Matrix

Grain Zn concentration was strongly and positively correlated with the root Zn (r = 0.552 **), stem Zn (r = 0.597 **), petiole Zn (r = 0.618 **), pod Zn (r = 0.641 **), and leaflet Zn (r = 0.655 **) concentration with a significant negative correlation with the root Cd (r = −0.341 **) and grain Cd (r = −0.384 **). It was also negatively correlated with the stem Cd (r = −0.193), petiole Cd (r = −0.188), pod Cd (r = −0.125), and leaflet Cd (r = −0.153), which emerged as not being significant. On the other hand, the grain Cd concentration was positively and significantly (*p* < 0.01) correlated with the Cd concentration in the roots (r = 0.882), stem (r = 0.889), petiole (r = 0.896), leaflet (r = 0.846), and pod (r = 0.861). This strongly implies that Cd accumulation in the grains increased when the concentrations of other tissues also rose (Table 2).

### 3.9. Translocation Factors (TFs) of Cadmium to Grains

**Figure 8:**
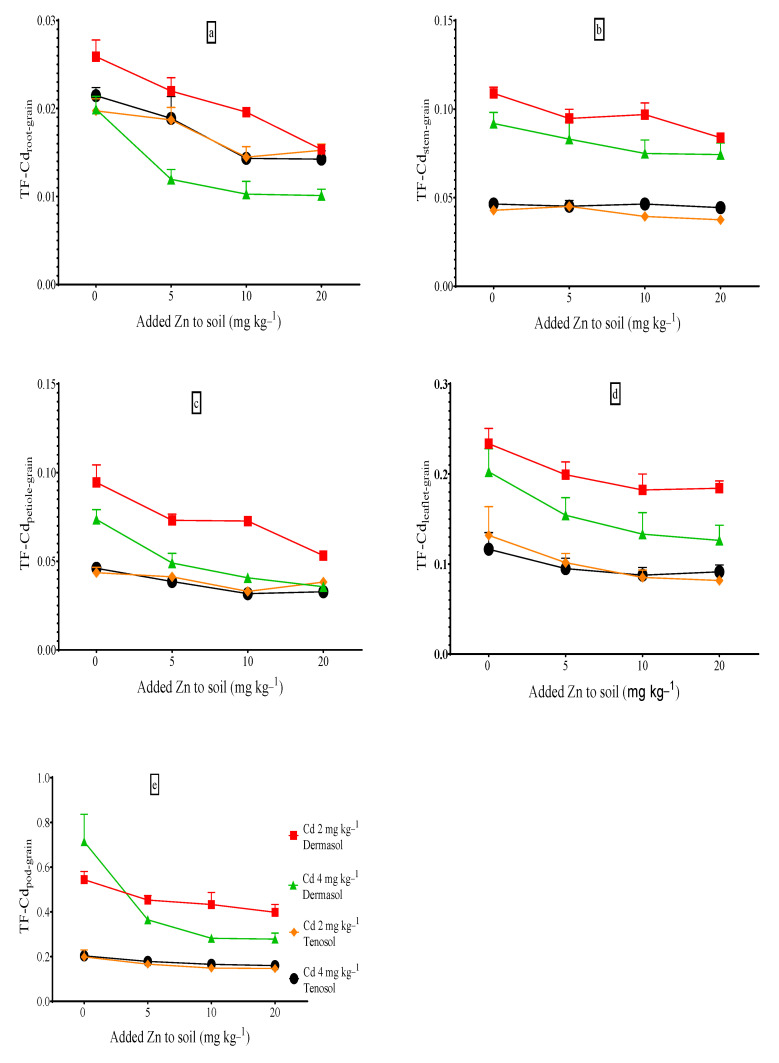
(**a**–**e**) Transfer of Cd to grains from other parts as affected by Zn, Cd, and soil types.

The TFs of Cd to the grains from the root (TFCd_root-grain_), stem (TFCd_stem-grain_), petiole (TFCd_petiole-grain_), leaflet (TFCd_leaflet-grain_), and pod (TFCd_pod-grain_) are presented in Figure 8a–e. A distinct variation was observed among the TFs of Cd to the grains from different parts for both soil types. For Dermosol, the TFCd_root-grain,_ TFCd_stem-grain,_ TFCd_petiole-grain,_ TFCd_leaflet-grain,_ and TFCd_pod-grain_ ranged from 0.030–0.047, 0.136–0.414, 0.115–0.184, 0.139–0.271, and 0.252–1.91, respectively, at the Cd control whereas, the values for Tenosol were 0.015–0.020, 0.019–0.022, 0.020–0.025, 0.067–0.074, and 0.158–0.177, respectively. The Cd translocation to the grains from other parts was higher for the Dermosol than the Tenosol. However, for the two soils, the maximum Cd transfer to the grains was observed from the pods followed by the leaflets, which indicates that Cd is mostly accumulated in the roots, stem, and petiole. A positive effect of the basal Zn supply was observed in the study, for example, added Zn restricted the upward movement of Cd so that the TFs of Cd to the grains from other parts demonstrated a downward trend (Figure 8).

### 3.10. Cadmium (Cd) Concentration in Pore Water

**Figure 9:**
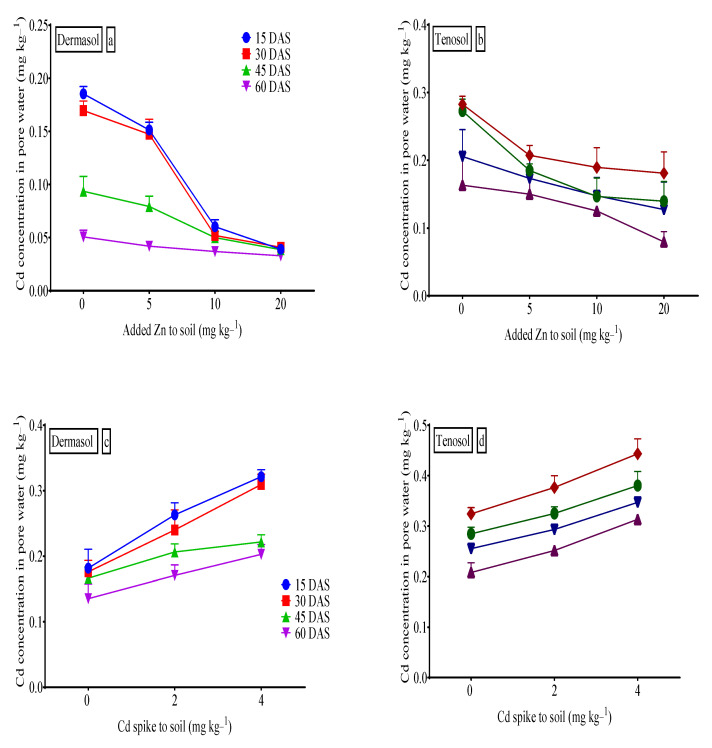
(**a**–**d**) Pore water Cd as affected by added Zn, Cd contamination to soils and days after sowing (DAS).

Cadmium concentrations in the pore water significantly differed (*p* < 0.01) for the added Zn and Cd contamination, soils, and days after sowing (DAS) (Figure 9). The Cd concentration (mg L^−1^) in the pore water reached the peaks of 0.185 and 0.324 for Dermosol and Tenosol, respectively, at 15 DAS, followed by a gradual slowdown as time passed. Presumably, Cd contamination in the soil significantly (*p* < 0.01) increased its presence in the pore water. The rise of Dermosol was 0.321 mg L^−1^ (76.4% higher) than 0.182 mg L^−1^ at 15 DAS, and this was due to the contamination of 4 mg of Cd kg^−1^ to the soil. It was 0.443 mg L^−1^ (36.7% higher) from 0.324 mg L^−1^ for the Tenosol. Zn supply to the soil types significantly (*p* < 0.01) reduced the availability of Cd in the pore water. The largest supply of Zn to the soil (20 mg kg^−1^) was responsible for the least Cd availability (0.039 mg L^−1^ at 15 DAS, about a 72% reduction compared to the control) for the Dermosol. However, for the Tenosol, at 15 DAS, the reduction in the Cd concentration was as low as 0.181 mg L^−1^ (about a 56.4% reduction compared to the control) from 0.283 mg L^−1^ for the same amount of Zn supply.

### 3.11. Health Risk Assessment of Cadmium Exposure

The Cd concentrations (mg kg^−1^) in the mung bean grains are presented in Table 3. Cd concentration in grains ranging 0.01 mg kg^−1^ to 0.1937 mg kg^−1^, irrespective of the added Zn and Cd contamination to the soils. The estimated daily intake (EDI) for children and adults was calculated based on the grain Cd concentrations in the mung beans grown in contaminated soils. For the Tenosol, the EDI (mg^−1^ kg^−1^ bw) of the grain Cd ranged from 0.84 × 10^−5^ (weekly 5.88 × 10^−5^) to 16.268 × 10^−5^ (weekly 113.9 × 10^−5^) and from 0.2689 × 10^−5^ (weekly 1.882 × 10^−5^) to 5.2075 × 10^−5^ (weekly 36.453 × 10^−5^) for children and adults, respectively. The highest EDI was recorded in the Cd spiked soil at 4 mg kg^−1^, which dropped to 13.72 × 10^−5^ (for children) and 4.392 × 10^−5^ (for adults) for the Tenosol due to the added Zn. On the other hand, for the Dermosol, an increasing Zn supply reduced the EDI from 10.136 × 10^−5^ to 6.664 × 10^−5^ and from 3.245 × 10^−5^ to 2.133 × 10^−5^ for children and adults, respectively.

We estimated THQ, the non-carcinogenic health risk of Cd consumption from mung bean grains, based on the EDI values. THQ was the lowest at the control for both soils but rose gradually with increasing Cd contamination in the soils. For the Tenosol, the highest THQ levels were 0.1627 and 0.0521 at 4 mg Cd kg^−1^ for children and adults, respectively. However, for the Dermosol, the THQ levels for children and adults were the highest at 2 mg Cd kg^−1^ contamination. For both soils, the added Zn to the soils reduced the THQ for children and adults. In this study, we measured ILCR for both children and adults when consuming mung beans. For children, the ILCR values ranged from 0.5292 × 10^−4^ to 10.249 × 10^−4^ and from 0.2646 × 10^−4^ to 6.3859 × 10^−4^ for the Tenosol and Dermosol, respectively. About one-third of the ILCR values were observed for adults in contrast to children. For both children and adults, the lowest ILCR value was observed at the control, and the highest was observed for the Tenosol when spiked with 4 mg of Cd kg^−1^. On the other hand, referring to the Dermosol, the smaller dose of Cd (2 mg kg^−1^) pushed up the ILCR to the peak.

## 4. Discussion

The interactions between Zn and Cd on mung beans were investigated in two soil types differing in DTPA-Zn to find out whether added Zn might remediate the damage caused by Cd. Zn and Cd partitioning were accomplished in mung bean tissues, such as the root, stem, petiole, leaflet, pod, and grain. Significant variations in metal accumulation were observed in different tissues due to the soil types and added Zn and Cd contamination to the soils. At control, the concentration of Cd followed the pattern, root > stem > petiole > pod > leaflet > grain, and those for root > stem > petiole > leaflet > pod > grain for the Dermosol and Tenosol, respectively (Figure 1). The sequential order of Cd in the plant was roots > leaves > fruits > grains [59], suggesting that only a small proportion of Cd was transported to the above-ground parts of the plants. In a hydroponic experiment with mung beans, Rashid et al. [60] found the highest Cd accumulation in the roots followed by the stem and leaves, i.e., Cd in the tissues followed the order, root < stem < leaves. In the soybean (*Glycine max* L.), it emerged that only 98% of the total Cd was taken up from the soil accumulates in the roots while the remainder was translocated to other parts by vascular bundles [61]. It indicates that Cd is translocated through the xylem in most plants and not readily translocated by phloem [62], although there are exceptions, e.g., where Cd concentration was higher in older leaves than the roots [63]. Due to direct contact, the roots generally contain a larger concentration of Cd, a mechanism through which plants protect their edible parts from contamination [64]. These may also include the co-precipitation of the metals in sections that are metabolically inactive [65].

In this study, irrespective of the presence of Cd, an increasing soil Zn supply consistently increased the Zn concentration in all the tissues, including grains, supporting agronomic biofortification [66]. This is also supported by the other literature, which shows that agronomic biofortification with Zn increases its accumulation [67,68,69] which results in the reduction of human Zn deficiency/malnutrition [70,71]. At the Zn control, its concentration in the grains was 24.33 and 25.04 mg kg^−1^ for the Dermosol and Tenosol, respectively. The added Zn (@5 mg Zn kg^−1^) to the Dermosol and Tenosol was responsible for the increment of Zn in the grains by about 52.5% (37.03 mg kg^−1^) and 54.7% (38.7 mg kg^−1^), respectively (Figure 1f). In a pot study [72], it was revealed that the grain Zn concentration in mung beans improved from 42.6 to 60.1 mg kg^−1^ due to various Zn application methods. Another study on mung beans revealed that the application of Zn as ZnSO_4_ increased grain Zn from 28.4 to 48 mg kg^−1^ [73]. It indicates that an external Zn supply improves Zn accumulation in plant parts, including in the grains [74,75,76,77], and the same was observed in this study. The presence of Cd diminished the accumulation of Zn in all tissues, including the grains (Figure 7b). Generally, Cd interferes with the translocation of calcium (Ca), phosphorus (P), magnesium (Mg), potassium (K), and manganese (Mn) [78], reducing iron (Fe) and Zn [79]. Cd also inhibits photosynthetic activities (chlorophyll a, b, total, and carotenoids); hence plants show toxicity symptoms [80], which lead to reduced growth and low Zn accumulation. A sharp escalation of Zn in the tissues was found due to the added Zn, and this has been supported by an investigation with green peas and beet [81]. Plants face a deficiency in essential elements, particularly Zn, for competition with Cd under conditions of Cd stress [65]. The translocation of Cd in grains from different parts of mung beans decreased with an increase in Zn supply, as reported in this study (Figure 7a,b). Thus, Zn biofortification through agronomic means serves two purposes simultaneously, firstly it enhances Zn accumulation in grains, and secondly, it excludes Cd in edible parts of the crops. The concurrent benefit of Zn biofortification to eradicate malnutrition and Cd remediation investigated by Kailasam and Peiter [82] supports our findings. Zn plays a vital role in Cd remobilization in plants and its translocation from the root to shoots and shoots to grains [83,84,85,86]. Zn supply may inhibit the loading of Cd in the phloem [84,87] and this, in turn, hinders the loading of Cd in the phloem, thus reducing Cd accumulation in the grains of plants. This may happen due to the competition between these two metals during their uptake at the root surface and translocation within plants. To keep plant growth, as well as to minimize Cd accumulation, plants have some protective mechanisms, such as Cd exclusion to upper tissues, the regulation of phytohormones (ethylene, jasmonic acid, salicylic acid, abscisic acid), and the activation of antioxidant enzyme activities (CAT, SOD, POD, MDA, etc.) [35,37,88,89].

However, the opposite results were found in mung bean tissues grown up to a vegetative stage in Hoagland’s nutrient solution, where the transport of Cd from the root to upward parts was in an upward trend despite the increasing Zn levels [60]. Being a divalent cation, Zn shares many transporters and binding sites with Cd; hence, an increase or decrease in Cd uptake happens in plants [83]. Irrespective of Zn and Cd treatments, the Tenosol demonstrated a larger accumulation of Cd in the tissues compared to the Dermosol. This might be due to the lower pH of Tenosol than Dermosol, which favored the higher Cd accumulation [61].

Soil pH is one of the main factors controlling the availability of Cd in plants, and a higher pH is commonly reduced by a Cd uptake in plants [61,90,91]. In this study, Dermosol was characterized by a higher pH (pH 7.7), and the grain Cd fell significantly when compared to the Tenosol (pH 6.4) due to the added Zn. The decreased grain Cd in the wheat due to Zn spray in the location of a high pH was also observed by Oliver et al. [21]. A recent study performed on rice varieties by Siddique et al. [92] supports our findings, where the author found that Cd accumulation in rice grains was less in soils with higher pH.

In our study, the highest Cd concentrations (mg L^−1^) in pore water were 0.185 and 0.324 for the Dermosol and Tenosol, respectively, at 15 DAS, followed by a gradual slowdown in the course of time (Figure 9). Wang et al. [93] examined lettuce and found 0.11 to 1.7 mg L^−1^ in the red soil and 0.06–1.3 mg L^−1^ in the yellow-brown soil, respectively, when the soil was spiked with five levels of Cd (0, 1, 2, 2.5, 5.0, 7.5, and 10 mg kg^−1^ soil) and in his experiment, the alleviated Cd levels enhanced the pore water Cd. Concentrations of Cd in the pore water were also matched with other amounts [94,95,96]. Over the course of time, Cd availability in the pore water gradually waned in our investigation. Similar findings were observed in rice [92], which also detected an enhanced Cd concentration (up to 76% compared to the control) in the pore due to Cd contamination in the soil.

The weekly tolerable Cd intake limit is 0.7 and 2.5 × 10^−3^ mg^−1^ kg^−1^ bw, respectively according to the US Department of Health and Human Services [97] and the European Food Safety Authority [98]. According to the joint FAO/WHO Expert Committee on Food Additives (JECFA), the provisional tolerable weekly intake (PTWI) value of Cd is 5.8 × 10^−3^ mg^−1^ kg^−1^ bw, (1 × 10^_3^ mg^−1^ kg^−1^ bw per day) (PTDI) [99]. Table 3 shows that the EDI values of Cd from mung bean consumption were below the provisional tolerable weekly intake (PTDI) irrespective of Cd contamination in both soils. The THQ values of mung bean grains grown in Cd spiked soils did not exceed the safe level (1.0), indicating there are no non-carcinogenic health risks when eating these grains. For Cd, the THQ values were 0.003 for adults, and 0.004 for children, based on the consumption of legumes [100].

The THQ of Cd for the consumption of cereals, legumes, and their products in Beijing, China, was 0.08 [101]. Due to the long-term consumption of 13 types of foodstuffs, including mung beans, the THQ value was 0.18 for Cd in China [102]. According to the US EPA, the tolerable limit of ILCR for regulatory purposes ranged from 1.0 × 10^−6^ to 1.0 × 10^−4^ [55]. The ILCR values of Cd exposure from mung bean grains were also estimated (Table 3). Our results showed that except for the control and sole Zn-treated plants, the ILCR values at all the treatment combinations were higher than the permissible limit of 1.0 × 10^−4^. This means that there is a potential for the carcinogenic risk of Cd through the consumption of mung beans. The ILCR value at Cd treatments had the highest risk in contrast to the amended ones.

## 5. Conclusions

Basal Zn supply favored the grain Zn biofortification as it increased its accumulation in all mung bean parts. It also decreased the Cd translocation to the grains to a remarkable degree. Zn at 5 mg kg^−1^ helped to boost grain Zn and mitigate Cd contamination in the grains. The consumption of the mung bean grains grown did not demonstrate any non-carcinogenic effects for both children and adults. This was evident in the EDI and PTDI values which were below the safety limit, although the ILCR values exceeded it, indicating the possibility of some carcinogenic effect. Added Zn helped reduce the non-carcinogenic and carcinogenic effects on humans. Further studies encompassing the Zn application protocols with other sources are warranted.

## Figures and Tables

**Table 1:** Physical and chemical properties of the soils.

Soil Types	pH (H_2_O)	EC µs/cm	%C	%N	%S	Texture	% Sand	% Silt	% Clay	Cd (mg kg^−1^)	Zn (mg kg^−1^)	DTPA Zn (mg kg^−1^)	As (mg kg^−1^)	Pb (mg kg^−1^)	Cr (mg kg^−1^)
Dermosol	7.7	161.2	2.7	0.21	0.055	Silt loam	33.8	55	11.2	0.01	5.9	0.5	1.6	5.2	9.1
Tenosol	6.4	190	1.4	0.22	0.009	Sandy loam	63.8	23.8	12.5	0.05	37	5	6.2	8.8	6.2

**Table 2:** Correlation among traits for Zn-Cd interactions (*n* = 24).

Parameters	Root Zn	Root Cd	Stem Zn	Stem Cd	Petiole Zn	Petiole Cd	Pod Zn	Pod Cd	Leaflet Zn	Leaflet Cd	Grain Zn	Grain Cd
Root Zn	1.00											
Root Cd	0.21	1.00										
Stem Zn	0.887 **	0.03	1.00									
Stem Cd	0.414 **	0.815 **	0.269 *	1.00								
Petiole Zn	0.808 **	−0.028	0.898 **	0.229	1.00							
Petiole Cd	0.403 **	0.913 **	0.226	0.925 **	0.156	1.00						
Pod Zn	0.801 **	0.035	0.842 **	0.281 *	0.862 **	0.235 *	1.00					
Pod Cd	0.441 **	0.829 **	0.266 *	0.927 **	0.219	0.949 **	0.281 *	1.00				
Leaflet Zn	0.746 **	−0.158	0.860 **	0.090	0.862 **	0.025	0.833 **	0.072	1.00			
Leaflet Cd	0.399 **	0.833 **	0.244 *	0.896 **	0.179	0.938 **	0.255 *	0.908 **	0.033	1.00		
Grain Zn	0.552 **	−0.341 **	0.597 **	−0.193	0.618 **	−0.188	0.641 **	−0.125	0.655 **	−0.153	1.00	
Grain Cd	0.165	0.882 **	−0.002	0.889 **	−0.058	0.896 **	−0.012	0.861 **	−0.188	0.846 **	−0.384 **	1.00

** Significant at *p* ≤ 0.01; * Significant at *p* ≤ 0.05.

**Table 3:** Health risk assessment for consumption of mung beans grown in Cd and Zn-treated soils.

Soil Types	Zn (mg kg^−1^)	Cd (mg kg^−1^)	Grain Cd (mg kg^−1^)	EDI × 10^−3^		THQ		ILCR × 10^−4^	
				Children	Adults	Children	Adults	Children	Adults
Tenosol	0	0	0.0100	0.0084	0.0027	0.0084	0.0027	0.5292	0.1694
	5	0	0.0107	0.0090	0.0029	0.0090	0.0029	0.5647	0.1807
	10	0	0.0103	0.0087	0.0028	0.0087	0.0028	0.5467	0.1750
	20	0	0.0103	0.0087	0.0028	0.0087	0.0028	0.5467	0.1750
	0	2	0.1667	0.1400	0.0448	0.1400	0.0448	8.8202	2.8234
	5	2	0.1633	0.1372	0.0439	0.1372	0.0439	8.6434	2.7668
	10	2	0.1413	0.1187	0.0380	0.1187	0.0380	7.4792	2.3941
	20	2	0.1377	0.1156	0.0370	0.1156	0.0370	7.2855	2.3321
	0	4	0.1937	0.1627	0.0521	0.1627	0.0521	10.2490	3.2807
	5	4	0.1717	0.1442	0.0462	0.1442	0.0462	9.0848	2.9081
	10	4	0.1690	0.1420	0.0454	0.1420	0.0454	8.9435	2.8628
	20	4	0.1633	0.1372	0.0439	0.1372	0.0439	8.6434	2.7668
Dermosol	0	0	0.0050	0.0042	0.0013	0.0042	0.0013	0.2646	0.0847
	5	0	0.0057	0.0048	0.0015	0.0048	0.0015	0.3001	0.0960
	10	0	0.0053	0.0045	0.0014	0.0045	0.0014	0.2821	0.0903
	20	0	0.0050	0.0042	0.0013	0.0042	0.0013	0.2646	0.0847
	0	2	0.1207	0.1014	0.0324	0.1014	0.0324	6.3859	2.0441
	5	2	0.1050	0.0882	0.0282	0.0882	0.0282	5.5566	1.7787
	10	2	0.1020	0.0857	0.0274	0.0857	0.0274	5.3978	1.7279
	20	2	0.0793	0.0666	0.0213	0.0666	0.0213	4.1981	1.3438
	0	4	0.1120	0.0941	0.0301	0.0941	0.0301	5.9270	1.8973
	5	4	0.1020	0.0857	0.0274	0.0857	0.0274	5.3978	1.7279
	10	4	0.0863	0.0725	0.0232	0.0725	0.0232	4.5686	1.4624
	20	4	0.0777	0.0652	0.0209	0.0652	0.0209	4.1103	1.3157

## Data Availability

The data that support this research will be shared upon reasonable request to the corresponding authors.
